# An improved *Plasmodium cynomolgi *genome assembly reveals an unexpected methyltransferase gene expansion

**DOI:** 10.12688/wellcomeopenres.11864.1

**Published:** 2017-06-16

**Authors:** Erica M Pasini, Ulrike Böhme, Gavin G. Rutledge, Annemarie Voorberg-Van der Wel, Mandy Sanders, Matt Berriman, Clemens HM Kocken, Thomas Dan Otto

**Affiliations:** 1 Biomedical Primate Research Centre, Rijswijk, Lange Kleiweg 161, 2288GJ Rijswijk, Netherlands; 2 The Wellcome Trust Sanger Institute, Hinxton, Cambridge, CB10 1SA, UK

**Keywords:** P. cynomolgi, PacBio assembly, P. coatneyi, methyltransferase

## Abstract

Background: *Plasmodium cynomolgi, *a non-human primate malaria parasite species, has been an important model parasite since its discovery in 1907. Similarities in the biology of *P. cynomolgi* to the closely related, but less tractable, human malaria parasite *P. vivax* make it the model parasite of choice for liver biology and vaccine studies pertinent to *P. vivax *malaria. Molecular and genome-scale studies of *P. cynomolgi* have relied on the current reference genome sequence, which remains highly fragmented with 1,649 unassigned scaffolds and little representation of the subtelomeres.

Methods: Using long-read sequence data (Pacific Biosciences SMRT technology), we assembled and annotated a new reference genome sequence, PcyM, sourced from an Indian rhesus monkey. We compare the newly assembled genome sequence with those of several other *Plasmodium* species, including a re-annotated *P. coatneyi* assembly.

Results: The new PcyM genome assembly is of significantly higher quality than the existing reference, comprising only 56 pieces, no gaps and an improved average gene length. Detailed manual curation has ensured a comprehensive annotation of the genome with 6,632 genes, nearly 1,000 more than previously attributed to *P. cynomolgi*. The new assembly also has an improved representation of the subtelomeric regions, which account for nearly 40% of the sequence. Within the subtelomeres, we identified more than 1300 *Plasmodium* interspersed repeat ( *pir*) genes, as well as a striking expansion of 36 methyltransferase pseudogenes that originated from a single copy on chromosome 9.

Conclusions: The manually curated PcyM reference genome sequence is an important new resource for the malaria research community. The high quality and contiguity of the data have enabled the discovery of a novel expansion of methyltransferase in the subtelomeres, and illustrates the new comparative genomics capabilities that are being unlocked by complete reference genomes.

## Introduction

*Plasmodium cynomolgi*, a non-human primate malaria parasite first mentioned by Mayer in 1907 ^1^ and established as a separate species from *P. inui* by Mulligan in 1935 ^2^, has been used as a model parasite species since its discovery. First used to establish the level of susceptibility of Malaysian Anophelines to non-human primate malaria ^3^, *P. cynomolgi* forms hypnozoites (a dormant liver stage), similar to those of human-infective *P. vivax* and *P. ovale* species. Other shared characteristics between *P. cynomolgi* and *P. vivax* include erythrocyte morphology (e.g. Schüffner's stippling), amoeboidity and the tertian periodicity of intraerythrocytic asexual development (48h life-cycle). *P. cynomolgi* is thus regarded as a powerful model for *P. vivax* and potentially *P. ovale* human malaria. The use of *P. cynomolgi* as a model organism is further reinforced by it being readily infective to and transmitted by a large number of mosquito species ^4– 7^, and by having a wide range of natural ^8– 10^ and experimental hosts ^3, 11^.

A particular strength of the *P. cynomolgi* system is access to chronic infections and to the developing and dormant liver stages in a parasite similar to *P. vivax*. An *in vivo*- *vitro* shuttle system for the study of *P. cynomolgi* liver stages ^12^ is being exploited to better understand hypnozoite biology using molecular tools and genome-scale approaches, which rely on the availability of a complete and well annotated *P. cynomolgi* reference ** genome sequence. However, the current *P. cynomolgi* B reference is very fragmented ^13^, and lacks large parts of the subtelomeric regions, thought to harbour genes involved in host-parasite interactions. Other closely related malaria parasite species have been sequenced, including *P. coatneyi* ^14^ which is closely related to *P. knowlesi*, and *P. simiovale* that was sequenced but never systematically assembled ^15^.

In this paper, we describe the improved genome sequence assembly of the *P. cynomolgi* M strain and compare it the genomes of five other *Plasmodium* species ( *P. vivax*, *P. falciparum, P. knowlesi, P. coatneyi, P. simiovale*) that infect humans or monkeys, to uncover similarities and differences that may inform future studies aimed at harnessing *P. cynomolgi* as a model for *P. vivax* human malaria.

## Methods

### Samples

DNA was obtained from a blood stage infection of an Indian rhesus macaque donor with *P. cynomolgi* M strain stocks originally provided by Dr. Bill Collins from the Center for Disease Control, Atlanta. After PlasmodiPur filtration, parasites were matured *in vitro* overnight. Parasites were purified over a 15.1% (w/v) Nycodenz gradient and DNA was isolated using the Gentra Puregene Blood kit (Qiagen) and processed according to the manufacturers’ instructions. The material was handled carefully in order to ensure the integrity of the DNA was maintained.

### Ethical approval

Ethical approval for the donor infection was provided under DEC750 following Dutch and European legislation in terms of animal experimentation. Prior to the start of the experiment, ethical approval for the donor monkey infection was provided by the local independent ethical committee, complying with Dutch law (BPRC Dier Experimenten Commissie, DEC; agreement number DEC# 750). The monkey was healthy as assessed by a veterinarian and as determined by clinical and hematological parameters measured before the start of the experiment. The experiment was performed according to Dutch and European laws. The Council of the Association for Assessment and Accreditation of Laboratory Animal Care (AAALAC International) has awarded BPRC full accreditation. Thus, BPRC is fully compliant with the international demands on animal studies and welfare as set forth by the European Council Directive 2010/63/EU, and Convention ETS 123, including the revised Appendix A as well as the ‘Standard for humane care and use of Laboratory Animals by Foreign institutions’ identification number A5539-01, provided by the Department of Health and Human Services of the United States of America’s National Institutes of Health (NIH) and Dutch implementing legislation.

The donor monkey ( *Macaca mulatta*, male, age 5 years, Indian origin) used in this study was captive-bred and socially housed. Animal housing was according to international guidelines for nonhuman primate care and use. Besides the standard feeding regime, and drinking water ad libitum via an automatic watering system, the animal followed an environmental enrichment program in which, next to permanent and rotating non-food enrichment, an item of food-enrichment was daily offered to the macaque. Monitoring of parasitemia was done by thigh pricks each time followed by a reward. The intravenous injection and large blood collection were performed under ketamine sedation, and all efforts were made to minimize any suffering of the animal. The monkey was daily monitored for health and discomfort. Immediately after taking blood from the monkey, the monkey was cured from malaria by intramuscular injection of chloroquine (7.5 mg/kg, on 3 consecutive days) and the absence of parasites was verified two weeks after treatment by microscopy of Giemsa stained slides of thigh prick blood of the monkey.

### Sequencing, assembly and annotation of P. cynomolgi

Genomic DNA was sheared into 250–350 base-pair fragments by focused ultrasonication (Covaris Adaptive Focused Acoustics technology (AFA Inc., Woburn, USA), and amplification-free Illumina libraries were prepared ^16^. Paired 76-base reads were generated on the Illumina GAII platform according to the manufacturer’s standard sequencing protocol.

We also generated a SMRTbell template library using the Pacific Biosciences issued protocol (20 kb Template Preparation Using BluePippin Size-Selection System). Five SMRT cells were sequenced on the PacBio RS II platform using P5 polymerase and the chemistry version 3 (C3/P5).

Raw sequence data were deposited in the European Nucleotide Archive under accession number ERP000298.

Sequence data from the SMRT cells were assembled with HGAP ^17^ (version 2.3.0), assuming an assembly size of 30 Mb. The resulting draft assembly was further improved using the IPA script ( https://github.com/ThomasDOtto/IPA), version 1.0.1. This script performs the following steps:

1) deletes small contigs,

2) identifies overlapping contigs with low Illumina coverage,

3) orders contigs against the P. vivax P01 reference using ABACAS2 ^18^ (version 1),

4) corrects errors with Illumina reads using iCORN2 ^19^ (version 0.95),

5) circularizes the two plastid genomes with Circlator ^20^ (version 0.12.0); and

6) renames the chromosomes and contigs.

Draft genome annotation was transferred from *P. vivax* P01 using RATT ^21^ (version 1), and supplemented with the output of the Augustus ^22^ gene finder, trained on *P. vivax* P01 as described in ^23^. This was followed by manual curation of the gene models in Artemis ^24^ (version from January 2015).

### Re-annotation of P. coatneyi

The published *P. coatneyi* genome assembly ^14^ (accession numbers CP016239 to CP016252 from NCBI) contains several large open reading frames that appear to correspond to coding sequences, especially in the subtelomeric regions. Using the reference gnomes of *P. vivax* P01 and *P. knowlesi,* we re-annotated *P. coatneyi* using Companion ^25^ (version 1.0.1). ** Default settings were used, with the exception of a cut-off of 0.2 for the “Augustus” parameter.

### Analysis of P. simiovale

Short reads of *P. simiovale* were obtained from the SRA ^15^ (accession number SRR826495). The reads were assembled with MaSuRCA ^26^ (version 2.1.0), improved with PAGIT ^27^ (version 1) and annotated with Companion ^25^ (version 1.0.1), reference *P. vivax* P01 and default settings.

### OrthoMCL

To identify orthologues, genes from the following eleven genome sequences were clustered using OrthoMCL ^28^ (version 1.4): the present *P. cynomolgi* M, *P. vivax* P01 ^29^, *P. falciparum* 3D7 ^30^, *P. reichenowi* CDC ^31^, the re-annotated *P. coatneyi*, the rodent malaria parasites ( *P. yoelii*, *P. chabaudi* and *P. berghei* ^32^), *P. knowlesi* ^33^, *P. malariae* and *P. ovale curtisi* ^34^. We used the May 2016 version of the genome annotations, taken from GeneDB ^35^. ** The amino acid sequences were compared using a BLASTp all-against-all, with an E-value cut-off of 1e-6. OrthoMCL version 1.4 was used, and a PERL script ascribed the gene functions to each gene ID.

### MSP analysis

All the genes annotated as ‘merozoite surface protein’ from *P. falciparum*, *P. reichenowi* CDC, *P. ovale curtisi*, *P. malariae*, *P. cynomolgi* M, *P. vivax* P01, *P. coatneyi* and *P. knowlesi* were selected and compared with a BLASTp (E-value 1e-6 -F F). The results were visualized with Gephi ^36^ (version 0.9.1). Genes that clustered together in that analysis were aligned with mafft ^37^ (version 7.205, parameter --auto). The alignment was trimmed with GBLOCKS ^38^ (version 0.91b) in Seaview ^39^ (version 4.6.1) and the tree was built with raxML ^40^ (version 8.0.24) using the PROTGAMMAGTR model and a bootstrap of 100. Visualization was done in FigTree ^41^ (version 1.4.2).

### Methyltransferases

Genes with the product ‘methyltransferase’ were all selected as nucleotide sequences. A selection of these genes, based on sequence similarity, was aligned with mafft. The phylogenetic tree was generated as the MSP tree, using the PROTGAMMAIGTR model. Potential transpososons were analysed with http://www.girinst.org ^42^ (using the RepbaseSubmitter section).

### PIR analysis

The amino acid sequences of the *Plasmodium* interspersed repeat ( *pir*) genes were extracted from five genomes (PcyM, *P. vivax* P01, *P. coatneyi*, *P. ovale curtisi* and *P. knowlesi*). First, low complexity sequences were trimmed with seg ^43^. Next, proteins smaller than 250aa were excluded. A BLASTp all-against-all comparison was run (E-value 1e-6, -F F, allowing for up to 4500 hits). The results were visualized in Gephi ^36^, clustered with the force field and the Reingold-Watermann algorithm. We also clustered the *pir* genes from the same BLAST with TribeMCL ^44^, using an inflation coefficient of 1.5.

## Results and discussion

### Improved genome assembly and annotation

**Figure 1.**
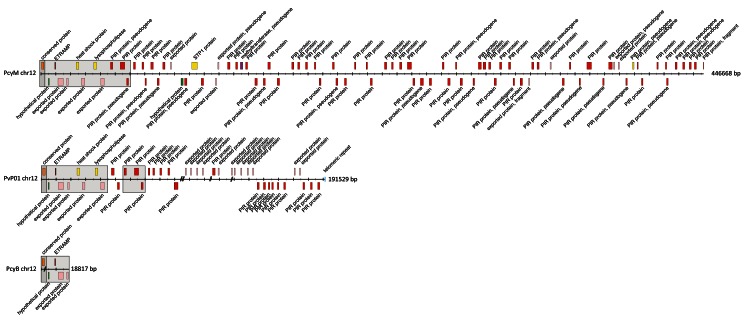
Organization of subtelomeric regions of chromosomes 12 of *P. cynomolgi* M, *P. vivax* P01 and *P. cynomolgi* B. The order and orientation of the genes in the subtelomeric region of chromosomes 12 (right hand side) of *P. cynomolgi* M (PcyM), *P. vivax* P01 (PvP01) and *P. cynomolgi* B (PcyB) are shown. Exons are shown in coloured boxes. // lines in PvP01 represent gaps. The dark shaded/grey areas mark the start of the conserved, syntenic regions to other *Plasmodium* species, e.g. *P. falciparum*. The lighter shaded/grey areas mark the syntenic regions between PcyM, PvP01 and PcyB.

The existing *P. cynomolgi* reference (B-strain, referred henceforth as PcyB) is highly fragmented, with 1,649 unassigned scaffolds. We generated a new reference genome sequence ( *P. cynomolgi* M strain – PcyM) using high-depth (>100x) Pacific Bioscience long-read sequence data and further improved it with Illumina sequencing reads. The new PcyM assembly is significantly larger than the PcyB assembly (31 versus 26 Mb) (see Table 1), more contiguous (N90 of 370kb versus 3.9kb), and has no sequencing gaps (0 versus 1943 gaps). The unassigned scaffolds have been reduced from 1,649 in PcyB to just 40 in the new PcyM assembly (see Figure 1).

**Table 1.** Comparison of *P. cynomolgi* M, *P. cynomolgi* B and *P. vivax* P01 genome features.

Genome features	PcyM	PcyB ^a^	PvP01 ^b^
**Nuclear genome**			
Assembly size (Mb)	30.6	26.2	29.0
Coverage (fold)	>150	161	212
G + C content (%)	37.3	40.4	39.8
No. contigs assigned to chrom.	14	14	14
No. unassigned contigs	40	1,649	226
# Sequencing Gaps	0	1943	560
No. genes ^c^	6,632	5,722	6,642
Average gene length (bp) ^d^	758	622	741
No. *pir* genes	1,373	265	1,212
**Mitochondrial genome** ^c^			
Assembly size (bp)	6,017	5,986	5,989
G + C content (%)	30.3	30,3	30.5
**Apicoplast genome**			
Assembly size (kb)	34.5	29.3	29.6
G + C content (%)	14.2	13.0	13.3
No. genes	30	23	30

^a,b^: Published sequences

^c^: Including pseudogenes and partial genes, excluding non-coding RNA genes.

^d^: Based on 1-1 orthologous

These improvements in contiguity and reduction of gaps had a large impact on the quality of the gene models. Overall, genes in PcyM are similar in size to their orthologues in *P. vivax* P01, while those in PcyB are around 20% shorter. In terms of annotation, 966 new genes were found in the PcyM assembly compared to PcyB, with most of these genes being found in the subtelomeres (see Table 2). The new genes, however, also include 119 genes that are 1-1 orthologous to genes in *P. vivax*. Due to the manual curation, 12% more genes have been assigned a gene function in the new assembly. These systematic improvements make the PcyM genome sequence a better reference for the community to use when studying the biology of *P. cynomolgi* and relapsing malaria parasites in general.

The genome sequences were obtained from samples that were originally described as being two different strains, Mulligan (M strain) and Bastianelli (B-strain). However, a genome-wide comparison of the gene repertoires reveals that 67% of the 1:1 orthologues are identical, which is much more than the number of identical genes observed (32%) between two *P. vivax* isolates (P01 versus C01). This is in line with the findings in the original publication describing the PcyB genome assembly ^13^, suggesting that the two strains are likely derived from the same isolate. This was further confirmed by a recent study that analysed the diversity of several *P*. *cynomolgi* isolates ^45^. Although the authors proposed to call the isolate M/B, we will use the M(ulligan) nomenclature for continuity.

**Table 2.** Number of gene members of different (subtelomeric) multigene families in the genomes of *P. cynomolgi* B, *P. cynomolgi* M, *P. vivax* P01.

Subtelomeric genes *				other (previous) names
	PcyM	PcyB **	PvP01 **	
**Gene family**				
PIR protein	1373	265	1212	vir-like, kir-like
tryptophan-rich protein	39	36	40	Pv-fam-a, TRAG, tryptophan-rich antigen
methyltransferase, pseudogene	36	26 ***	0	
lysophospholipase	8	9	10	PST-A protein
STP1 protein	51	3	10	PvSTP1
early transcribed membrane protein (ETRAMP)	9	9	9	
Plasmodium exported protein (PHIST), unknown function	54	48	84	Phist protein (Pf-fam-b), RAD protein (Pv-fam-e)
reticulocyte binding protein	6	8	9 **	reticulocyte- binding protein, RBP
exported protein ****	276	175	447	

Key:

*Numbers including pseudogenes and partial genes

**Published sequence

***annotated as hypothetical protein

****ExportPred

### OrthoMCL clustering

**Figure 2.**
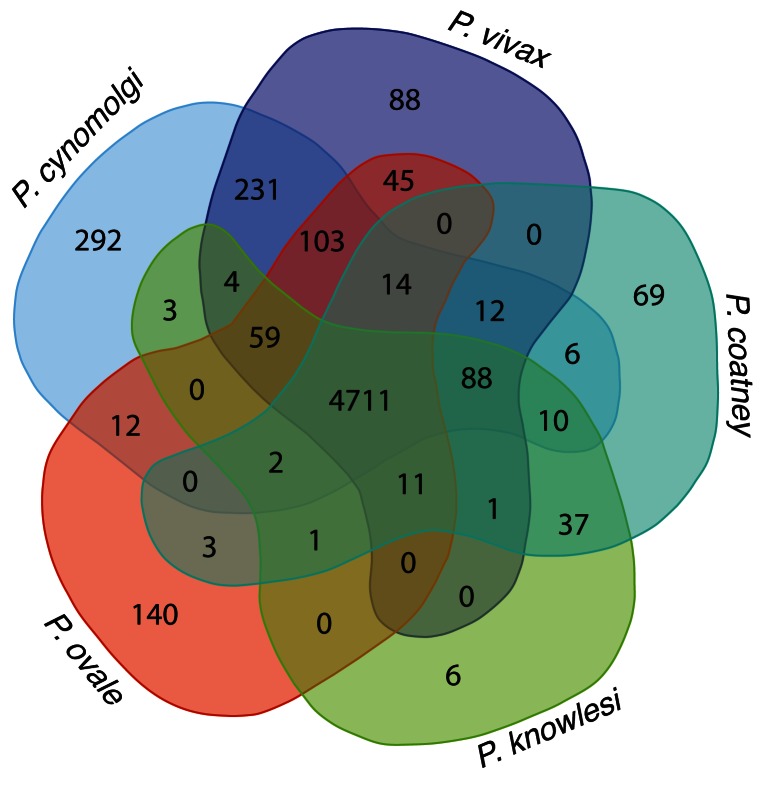
Orthologous classes of five genomes. Shared orthologous clusters produced using OrthoMCL version 1.4 with default parameters. The high number of shared clusters between species confirms the number of shared genes between the species. The 242 clusters between *P. vivax* and *P. cynomolgi* emphasise that they are closer related. The 103 clusters shared between *P. ovale*, *P. vivax* and *P. cynomolgi* might give insight into genes associated to the hypnozoite stages.

To look for conserved orthologues between species, an OrthoMCL ^28^ clustering of genes from eleven genome assemblies was performed (see Methods and Supplementary Table 1). We used the clustering to look further into genes potentially involved in the formation and development of the dormant hypnozoite stage. There are 103 gene clusters (see Figure 2) that are common to the relapsing parasites, but absent in *P. knowlesi* and *P. coatneyi*. Of these, 73 gene clusters are uniquely shared between *P. vivax* P01, PcyM and *P. ovale curtisi* GH01. The remaining 30 clusters are either shared with various combinations of the other nine parasite species (see Supplementary Table 1) or only with *P. malariae* (20 out of the 30 clusters).

The 73 clusters unique to the relapsing parasites include three tryptophan rich protein clusters where the orthology is 1:1:1 with the exception of one cluster in which *P. vivax* presents an expansion to four genes; two PHIST proteins (before named RAD and Pv-fam-e) clusters containing 1:1:1 orthologs; 11 clusters featuring 1:1:1 orthologs annotated as ‘Plasmodium exported proteins’; three clusters of 1:1:1 hypothetical protein orthologs; one cluster annotated as MSP-7 or MSP-7-like and 56 *pir* gene clusters showing different degrees of expansion in the three relapsing species. While their specificity is interesting, clusters corresponding to multigene families are probably less likely to have a direct function in dormancy. The hypothetical protein clusters (PcyM_0326800, PcyM_0423700 and PcyM_0904700), however, being specific to the three relapsing *Plasmodium* species, are intriguing, as is the MSP-like protein cluster.

**Figure 3.**
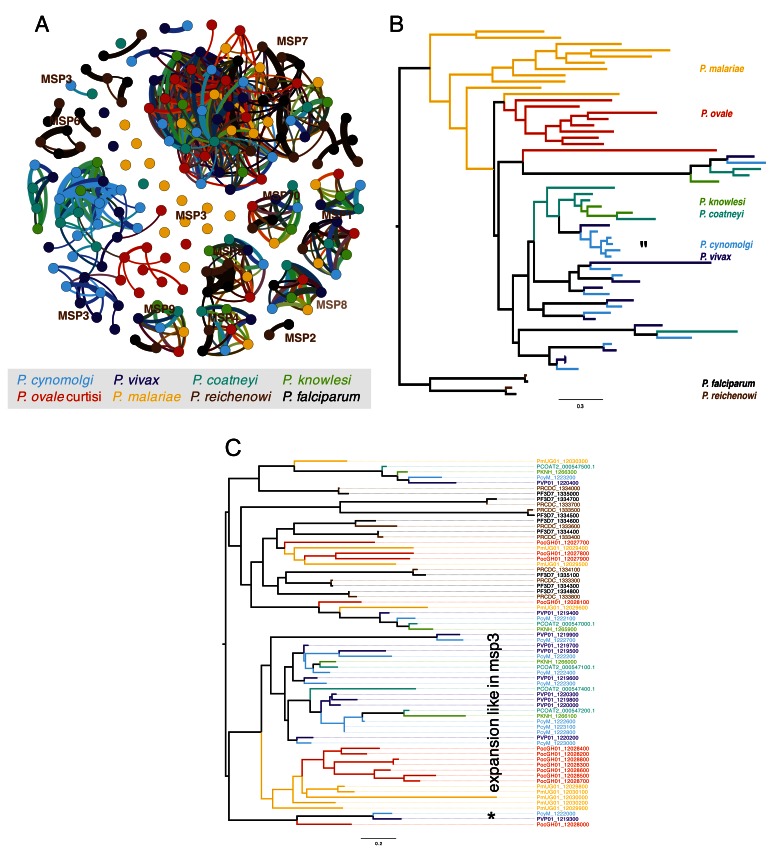
Analysis of expansion merozoite surface proteins. ( **A**) BLAST-based graph of all the merozoite surface proteins (MSP), (cut-off 20% global similarity). The different MSP types form clusters, apart from MSP3 which seem to be more diverse. ( **B**) Maximum likelihood tree (PROTGAMMAJTTF model, bootstrap at all branches in 100) of MSP3 and 2 laverania MSP6, shows species complex specific expansions in some species. The expansion was pre-speciation of *P. cynomolgi* and *P. vivax*. We also observe a MSP3 expansion in *P. cynomolgi*. “*” indicates an expansion of MSP3 in *P. cynomolgi*. ( **C**) As in ( **B**), but with MSP7 and MSP7. The tree is more complex, showing different types of MSP7. Some clades have a similar structure to MSP3, with specific expansions. “*” highlights a cluster containing MSP7 from parasites that have the hypnozoite stage.

***Paralogous expansion of the merozoite surface protein (MSP) family***. Although the specific function of the different merozoite surface proteins (MSPs) remains elusive, MSP-1 and MSP-3 are currently under evaluation as vaccine candidates. The OrthoMCL clustering shows that MSP-1, MSP-1 paralog, MSP-4, MSP-5, MSP-9 and MSP-10 are highly conserved and present across different *Plasmodium* species. MSP-2 and MSP-6 are present only in *P. falciparum* and *P. reichenowi* (see Figure 3A). In contrast, MSP-3 and MSP-7/7-like are highly expanded. MSP-3 is expanded in *P. vivax*, *P. malariae*, *P. ovale* and *P. cynomolgi* (see Figure 3B). Interestingly, while in *P. malariae* and to *P. ovale*, MSP-3 paralogs seem to be species-specific, in *P. cynomolgi, P. vivax*, *P. coatneyi* and *P. knowlesi* many of the paralogs seem to predate speciation, indicating that MSP-3 duplicated in the common ancestor of the latter four species. These findings of MSP-3 expansions are in line with the finding of multi-allelic diversification reported previously ^46^, but also confirm the expansion in *P. malariae* and *P. ovale*. In addition to the pre-speciation expansion in *P. cynomolgi*, a species-specific expansion of MSP-3 (see area indicated with ‘*’ in Figure 3B) genes suggests ongoing evolutionary pressure on these genes.

We also observed an expansion of MSP-7/7-like genes. In the OrthoMCL clustering, the genes were distributed in nine different clusters: 108, 4913, 5404, 5550, 5065, 6376 and 5765–5767 ( Supplementary Table 1). A phylogenetic tree of the MSP-7/7-like proteins revealed a complex evolutionary relationship (see Figure 3C), splitting the tree into three major clades. Across the tree we find paralogous expansions of different ages, some of which predate speciation. A particularly striking branch comprises only genes from the three hypnozoite-forming species. As a result of the large amount of genome sequences now available for different *Plasmodium* species, a complex pattern now emerges in the MSP7/7-like tree, suggesting that the different MSP7 proteins likely have different functions.

### Improved sub-telomeres reveals insights into subtelomeric gene families

The new high-quality PcyM assembly has an improved representation of the subtelomeric regions of the genome, which now encompass nearly 40% of the genome sequence. Manual curation of the gene annotation enabled the complete set of subtelomeric genes to be resolved (see Table 2). In *P. vivax,* genes encoding the exported protein family ‘PHIST’, and exported proteins in general (as predicted by ExportPred ^47^), have paralogously expanded compared to *P. cynomolgi* (84 vs 54). It is tempting to speculate about the reason for the higher number of exported proteins in *P. vivax*. One hypothesis is that it could be due to differences in the blood cells of humans compared to primates; while another could be that they are involved in the regulation of genes involved in host parasite interaction. In *P. falciparum,* it was suggested that PHISTb regulates *var* genes ^48^. In *P. cynomolgi*, we observed an expansion of the STP1 family (51 genes). STP1 proteins are common in *P. malariae* and *P. ovale curtisi* (166 and 70 genes, respectively), but are contracted in number in *P. vivax* (10 genes). One may also speculate that the expansion of PHIST and exported proteins in *P. vivax* compensates for the lack of STP1 proteins.

**Figure 4.**
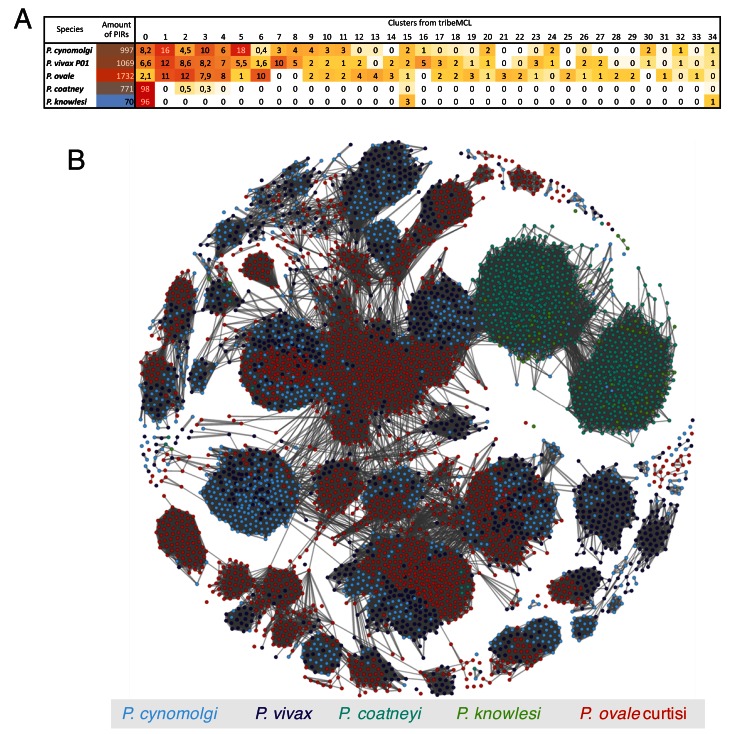
Cluster analysis illustrating the relatedness between the PIR proteins in five genomes. ( **A**) Classification (generated with tribeMCL inflation parameter 1.5) highlights the different types of PIR clusters. *P. coatneyi* and *P.* knowlesi. ( **B**) Network illustrating the relatedness of PIR between *P. ovale* (red), *P. cynomogli (*light blue) and *P. vivax* (blue), and between *P. coatneyi* (light green) and *P. knowlesi* (green). *P. ovale, P. cynomogli* and *P. vivax* clearly share the same general topology of PIR architecture. In contrast, *P. coatneyi* and *P. knowlesi* have a reduced number PIRs and also of PIR classes.

The largest multigene family in *P. cynomolgi* comprises *pir* genes. The *pir* superfamily occurs in all *Plasmodium* species ^49^, but their function remains poorly understood. Recent studies suggest a possible role in the regulation of the establishment of chronic infections ^50^ and they have been found expressed in liver stage infections of rodent parasites ^51^. An extensive repertoire of 1373 *pir* genes was identified in the PcyM assembly, compared to 263 in PcyB. This updated number puts the *P. cynomolgi pir* gene repertoire at a similar size to that of *P. vivax* (1,216), while *P. ovale curtisi* has an even larger repertoire (1,949). Conversely, *P. knowlesi*, has only 70 *pir* genes present. Interestingly, the re-annotated *P. coatneyi* genome that clusters closely to *P. knowlesi* has 827 *pir* genes (see Figure 4B). In the published annotation it has just 256 *pir* genes.

As previously reported ^29, 52^, the *pir* genes can be grouped based on sequence similarity. We observe that the diversity of the *pir* repertoire is dramatically reduced in *P. coatneyi* and *P. knowlesi*. Most of the *pir* genes form the same cluster (cluster 0; Figure 4A). However, that cluster splits into two groups in the gene-gene network due to the different lengths of the *pir* genes in *P. coatneyi* and *P. knowlesi* (see Figure 4B). One hypothesis for the loss of other *pir* types might be the occurrence of sicaVAR genes in *P. knowlesi* and *P. coatneyi* ^33^. The reduction of the *pir* repertoire is an interesting parallel to the *Laverania*, where the amount of *rif* genes (analogous to *pir* genes) is reduced but a new gene family evolved, the *var* genes. Additionally, in the *Laverania* the number of *rif* genes drops further when the parasite is in the human compared to the primate ^31^.

As for the other clusters, it seems that the underlying structure of the *pir* genes predates the speciation of *P. ovale*, *P. vivax* and *P. cynomolgi*. Depending on the type of *pir*, the amount can fluctuate, as can be seen by the large variance in number of genes per cluster. Some clusters are specific to *P. ovale* and some others contain just the two human malaria parasites, *P. vivax* and *P. ovale*. Interestingly, several *pir* genes have 1:1 orthologues across the different species ( Supplementary Table 1, see Figure 4B). As those genes seem to be conserved across evolutionary time, it is unlikely that they are extracellular (where they would be under immune pressure), rather they must have more conserved core functions.

**Figure 5.**
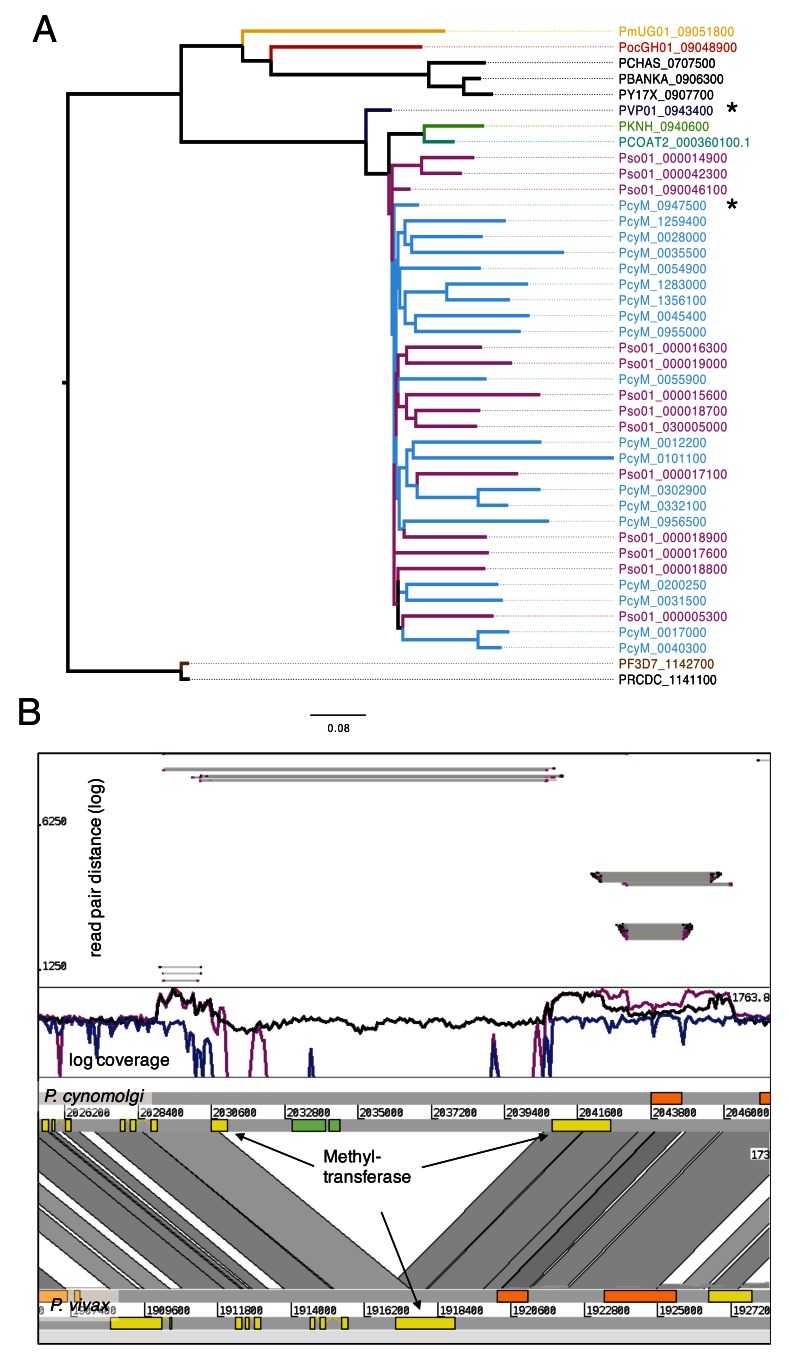
Expansion of Methyltransferase in *P. cynomolgi* and *P. simiovale*. ( **A**) Tree of methyltransferase in Plasmodium, including the expansion of those genes in *P. cynomolgi* (36) and *P. simiovale* (at least 15). The closest core genes are PVP01_0943400 and PcyM_0947500. ( **B**) Comparative view of *P. cynomolgi* and *P. vivax* on the locus of methyltransferase (*) of panel A. Interestingly, the locus in *P. cynomolgi* has an insertion with a subtelomeric gene that has a weak hit with to a putative DNA translocase Ftsk domain. Coverage plot mapped from *P. cynomolgi* reads (black), *P. vivax* (blue) and *P. simiovale* (magneta) is shown in log scale on *P. cynomolgi*. The methyltransferases are duplicated more than 35 times. As the height is roughly similar between the two duplications, we expect around the same number of methyltransferases in *P. simiovale* than in *P. cynomolgi*. The insert of the green gene is found just in *P. cynomolgi,* due to the missing coverage. The upper panel shows the distance of read pairs; the insertion of the region probably occurred after the duplication of the gene into the subtelomeres, as all reads from the duplications are connected over the insertion. The next core gene is also duplicated.

***Expansion of methyltransferases***. While paralogous expansions of *pir* genes and genes encoding MSP genes have been described in other *Plasmodium* species, *P. cynomolgi* exhibits an unexpected expansion of 36 methyltransferase pseudogenes. These pseudogenes are found in the subtelomeres, and were annotated as encoding 26 hypothetical proteins in the PcyB assembly. The role of pseudogenes in *Plasmodium* is little understood, but in several malaria parasite species conserved pseudogenes are found in the subtelomeres. In the OrthoMCL clustering, all 36 methyltransferase pseudogenes cluster with one full-length core gene (PcyM_0947500, Figure 5A). This gene is found on chromosome 9 and has one conserved orthologue across all other *Plasmodium* species (cluster 51, Supplementary Table 1), and is found in many other species on OrthoMCL as cluster OG5_129798. The 36 copies are spread evenly throughout the subtelomeres, without evidence of spatial clustering.

The methyltransferase pseudogenes contain motifs of the Caulimovirus, a virus often found integrated in to plant genomes, and of different retrotransposons families such as *aedes aegypti*, Gypsy, Helitron-5, CACTA-1, RTEX and CR1 (see Supplementary Table 2). While the Caulimovirus insert was mostly found to have occurred in an antisense orientation hinting towards a role in stability, the LTR and non-LTR insertions were found most often to have occurred in a sense orientation ^53^. The hits were mostly to low complexity regions, suggesting that recombination in the subtelomeres may be a result of mechanisms similar to those used by retro elements.

We also found evidence that this duplication of methyltransferases was also found in *P. simiovale,* a close outgroup to *P. cynomolgi*, *P. vivax*, and *P. knowlesi*. Fewer copies were observed in the *P. simiovale* assembly (13), but this may be due to the fragmentation of the assembly. Although they are generally less degenerate at their 5’ ends, they are nevertheless pseudogenized.

To further understand the duplication, we mapped the reads of *P. cynomolgi*, *P. simiovale* and *P. vivax* P01 against the locus on chromosome 9 containing the ancestral methyltransferase in *P. cynomolgi* (see Figure 5B). Although the coverage is shown as log scale, the coverage across the methyltransferase seems to be identical for *P. simiovale* and *P. cynomolgi*, but significantly lower for *P. vivax.* This leaves us to speculate that the number of methyltransferases is roughly the same in both *P. simioval*e and *P. cynomolgi*. Further, the coverage plot also reveals that the next core gene of unknown function, PcyM_0947600, is also duplicated. In PcyM we find two further paralogous genes: PcyM_0054800 PcyM_0012100. Furthermore, it is more often duplicated in *P. simiovale*, as the coverage of that gene is high ( Figure 5B). A search for structural similarity using I-TASSER ^54^ yielded no conclusive results.

A phylogenetic tree (see Figure 5A) shows the methyltransferase paralogs in *P. cynomolgi* and *P. simiovale* compared to the orthologues in the other species. The genes generally follow the species tree, but they are expanded in *P. cynomolgi* and *P. simiovale*. As *P. simiovale* is thought to be an outgroup to *P. cynomolgi* and *P. vivax,* we expect that P *. vivax* has lost the expansions.

We compared the location of the ancestral methyltransferase between PcyM and *P. vivax*. To our surprise, we found a potential open reading frame inserted between two methyltransferases in *P. cynomolgi*. A tBLASTn of that CDS against the Nucleotide NCBI database revealed no significant similarity to any other sequence, except for the subtelomeres of *P. vivax* and *P. cynomolgi*. A very weak hit (e-value of e-4) to a DNA translocase FtsK, is an interesting finding, in light of the potential LTR transposon-like sequences discussed previously, but is to be taken with caution. This particular open reading frame is absent in *P. simiovale* and seems that have occurred subsequent to the expansion and is not likely to be implicated in the expansion itself.

It remains speculative if the paralogs of the methyltransferase genes and the adjacent gene were functional in the ancestor. Hypothetical roles of the methyltransferase could involve any of the following: 1) the epigenetic control of ** differential *pir* gene expression in acute and chronic infections ^50^, 2) the sequence may have a role in genome stability and recombination, or 3) this could be a selfish gene that was able to transpose.

### Conclusion

The availability of a new and improved *P. cynomolgi* reference genome sequence will enable in-depth studies of this widely used model parasite, including investigations into dormant stages and the selection of new drug targets and vaccine candidates. High quality genomics related studies will now be possible, including studies of previously missed core genes. In particular, the improved subtelomeres have enabled us to dissect the *pir* gene family further, and have revealed a novel and unexpected expansion of methyltransferase genes.

## Data and software availability

The project number of the *P. cynomolgi* raw reads is deposited in the European Nucleotide Archive under accession number ERP000298. The submitted genome is under the project number PRJEB2243.

The chromosomes have the accession: LT841379-LT841394, and the scaffolds: FXLJ01000001-FXLJ01000040.

The annotation can be found at: ftp://ftp.sanger.ac.uk/pub/project/pathogens/Plasmodium/cynomolgi/M/Jan2017/

The automated re-annotation of *P. coatneyi* and the draft assembly of *P. simiovale* can be found at: ftp://ftp.sanger.ac.uk/pub/project/pathogens/Plasmodium/coatneyi/ReAnnotation/ and ftp://ftp.sanger.ac.uk/pub/project/pathogens/Plasmodium/simiovale/May2017/, respectively.

The IPA software is available on GitHub: https://github.com/ThomasDOtto/IPA. Version 1.0.1 was used for this work.

The software is also available on Zenodo: https://doi.org/10.5281/zenodo.806818 ^55^

License: GNU General Public License v3.0

## Supplementary material

**Supplementary Table 1: Annotated OrthoMCL of 11 species**.

**Supplementary Table 2: Results of the search for motifs associated with transposons, http://www.girinst.org**.
