# Supplementary material for: An improved Plasmodium cynomolgi genome assembly reveals an unexpected methyltransferase gene expansion
Source: Wellcome Open Res. 2017 Jun 16;2:42. [Version 1] doi: 10.12688/wellcomeopenres.11864.1 (PMC5500898; doi:10.12688/wellcomeopenres.11864.1)
Supplement: Peer review history

# Peer review: Referee response for version 1

**DOI:** 10.21956/wellcomeopenres.12821.r23586

**Published:** 2017-06-30

**Authors:** Richárd Bártfai

**Affiliations:** 1 Department of Molecular Biology, Radboud University, Nijmegen, Netherlands

In this study, Pasini and colleagues improved the assembly and annotation of the *P. cynomolgi* genome. They performed short-read Illumina and long-read PacBio sequencing of DNA isolated from the PcyM strain. Assembly of these sequences yielded a markedly improved reference genome with substantially less gaps and much better coverage of the subtelomeric regions. Furthermore, manual curtation resulted in substantially improved gene models and better annotation of gene functions. Comparative genome analysis highlighted interesting dynamics in copy number variation of specific gene families (MSP, STP1, PIR), and in particular a peculiar expansion of methyltransferase pseudogenes.

 This manuscript is well written and describe a well-executed assembly and annotation of the *P. cynomolgi* reference genome. This reference genome will be well appreciated in the field and will likely fuel further exploration of genome evolution, vaccine candidates and hypnozoite biology alike.

 “Major” comments:

- It would be important to clarify what is the relevance for the use of the *P vivax* reference genome during the assembly. I.e. are there contigs which are purely linked based on their assumed synteny to *P. vivax*. If so how many such “connections” are present? Also a more detailed description of the manual annotation would be appreciated. I.e. what sort of changes has been made and based on what kind of evidences?
- I think it would be important to clarify if the PcyB and M strains are indeed represent one and the same (in which case the PcyB/M name would be appropriate) or two closely related isolates. If it can be concluded with high confidence this information should be mention in the abstract as well.
- The potential function and origin of the methyltransferase could perhaps be better analyzed and discussed. After a quick domain search I realized that this methyltransferases also contain multiple ankyrin domains. More importantly homology search suggests that this is/was a nicotinamide N-methyltransferase and hence might also play a role in nicotinamide metabolism. Intriguingly some methyltransferases (e.g. SET8, Kishore, BMC Evol Bio, 2013 ^1^) and some members of the nicotinamide pathway (O’Hara, PLOS One, 2014 ^2^) in Plasmodia might be resulted from horizontal gene transfer. Therefore, it could be interesting to investigate if this could be the case for this particular gene as well.

Minor points:

- Supplementary table 1 is not very useful in its current form. It is rather cumbersome to select out the genes which are only present in certain species. Perhaps the authors could sort the table according to the clusters presented on figure 2.
- It is nice that the same color scheme is used throughout the manuscript, but the color of Pcy, Pc and Pk are rather similar and difficult to tell apart. In particular on Figure 4 this is problematic.
- Perhaps it would be more logic to discuss the improved subtelomeres earlier in the manuscript (i.e before the OrthoMCL clustering)
- Figure 3 B and C could be better labelled.
- The improved reference genome should be made available in GeneDB and PlasmoDB as well.
- Finally, the authors mention that genes specific to hypnozoite forming parasites mainly belong to variant multigene families and unlikely to be relevant for hypnozoite formation. Perhaps it would be worthwhile identifying genes which are not unique, but substantially different in these parasites (indels, unexpectedly high number of SNPs, etc).

I have read this submission. I believe that I have an appropriate level of expertise to confirm that it is of an acceptable scientific standard.

## References

[1] KishoreSPStillerJWDeitschKW: Horizontal gene transfer of epigenetic machinery and evolution of parasitism in the malaria parasite Plasmodium falciparum and other apicomplexans. *BMC Evol Biol*.2013;13: 10.1186/1471-2148-13-37 37 10.1186/1471-2148-13-37 23398820PMC3598677

[2] O'HaraJKKerwinLJCobboldSATaiJBedellTAReiderPJLlinásM: Targeting NAD+ metabolism in the human malaria parasite Plasmodium falciparum. *PLoS One*.2014;9(4) : 10.1371/journal.pone.0094061 e94061 10.1371/journal.pone.0094061 24747974PMC3991606

# Peer review: Referee response for version 1

**DOI:** 10.21956/wellcomeopenres.12821.r23884

**Published:** 2017-06-29

**Authors:** Aaron Jex

**Affiliations:** 1 Population Health and Immunity Division, Walter and Eliza Hall Institute of Medical Research, Melbourne, Vic, Australia

The current manuscript describes the resequencing and finishing to near chromosomal completion of the *Plasmodium cynomolgi* genome using high coverage PacBIO sequencing, the reannotation of the parasite's ~6,500 coding gene models and a detailed comparative analysis of its major gene families relative to related primate clade species of *Plasmodium*, including *P. vivax, P. ovale* and *P. knowelsi* as well as a draft assembly and annotation of *P. simiovale *and a reannotation of *P. coatneyi*. As the authors describe, *P. cynomolgi *is an important model for *P. vivax, *a relapsing human infectious species that now predominates as the primary cause of malaria in the Asia-Pacific and Americas. The authors correctly note that the current draft assembly the *P. cynomolgi *genome, which was produced several years ago, is highly fragmented, incomplete and has truncated / inadequate gene models. Though of an acceptable standard at the time, it is clear that this prior genome is no longer adequate to act as a reference of *P. cynomolgi *research. Based on this, the current manuscript is a timely contribution that will provide an excellent resource for the malaria research community. The paper itself is well written, the figures are very nicely conceived and presented and the methods used are appropriate and expertly applied. I have no hesitation in recommending this study for publication.

 Minor comments:

1. 'Anophelines' shouldn't be capitalized (paragraph 1 of the introduction)
2. 'Reference gnomes' under 'Re-annotation of P. coatneyi' should be 'Reference genomes'
3. It would be interesting to know if any of the *P. cynomolgi *genes represented by the 103 ortholog clusters unique to *P. cynomolgi*, *P. ovale *and *P. vivax *are represented in the recent liver-stage transcriptome (particularly the hypnozoite transcriptome) published by Cubi *et al *( https://www.ncbi.nlm.nih.gov/pubmed/28256794). I wonder if this might be considered by the authors as a minor addition?
4. Could the authors please provide a more detailed explanation either in their methods section or in the results section for how they define the expanded methyltransferases they identify in *P. cynomolgi *as pseudogenes? I wonder if this will otherwise not be immediately clear to the reader.
5. Under the 'Paralogous expansion of ... MSP' section - 'while in *P. malaria *and to *P. ovale*' - I assume 'to' should be deleted here.
6. In Figure 3C, should this be 'expansion in msp3-like'? If not, could the authors explain what they mean by 'expansion like'?

I have read this submission. I believe that I have an appropriate level of expertise to confirm that it is of an acceptable scientific standard.
